# A case of bilateral human herpes virus 6 panuveitis with genomic viral DNA integration

**DOI:** 10.1186/s12348-014-0016-x

**Published:** 2014-06-19

**Authors:** Jasmina Bajric, Wendy M Smith

**Affiliations:** 1 Department of Ophthalmology, Mayo Clinic, 200 First Street SW, Rochester 55905, MN, USA

**Keywords:** Chromosomal integration, Human herpes virus-6, HHV-6, Immunocompromise, Panuveitis, PCR

## Abstract

**Background:**

We report a rare case of bilateral panuveitis from human herpes virus 6 (HHV-6) with genomic viral DNA integration in an immunocompromised man.

**Findings:**

A 59-year-old man with history of multiple myeloma presented with altered mental status, bilateral eye redness, and blurry vision. Examination revealed bilateral diffuse keratic precipitates, 4+ anterior chamber cell, hypopyon, vitritis, and intraretinal hemorrhages. Intraocular fluid testing by polymerase chain reaction (PCR) was positive for HHV-6. The patient was successfully treated with intravitreal foscarnet and intravenous ganciclovir and foscarnet. Despite clinical improvement, his serum HHV-6 levels remained high, and it was concluded that he had HHV-6 chromosomal integration.

**Conclusions:**

HHV-6 should be considered in the differential for infectious uveitis in immunocompromised hosts who may otherwise have a negative work-up. HHV-6 DNA integration may lead to difficulties in disease diagnosis and determining disease resolution.

## Findings

Human herpes virus-6 (HHV-6) is a ubiquitous virus that infects most children by the age of three years. While the seroprevalence in the adult population approaches 95%, and HHV-6 reactivations are known to be common after organ transplantation, clinical disease is rare after the primary infection [1]. Although HHV-6 is closely related to cytomegalovirus (CMV), ocular disease due to HHV-6 has been described in very few patients [2-8]. We report the case of an immunocompromised man who presented with encephalitis and severe bilateral panuveitis as a result of HHV-6 reactivation. Integration of the viral genome into the host DNA, a unique characteristic of HHV-6, complicated the clinical management of our patient.

### Case report

A 59-year-old man with a history of multiple myeloma status post allogeneic stem cell transplant was admitted to our hospital with fevers and a soft tissue infection. On the fourth day of hospitalization, he developed a headache, somnolence, bilateral eye redness, and blurred vision.

**Figure 1:**
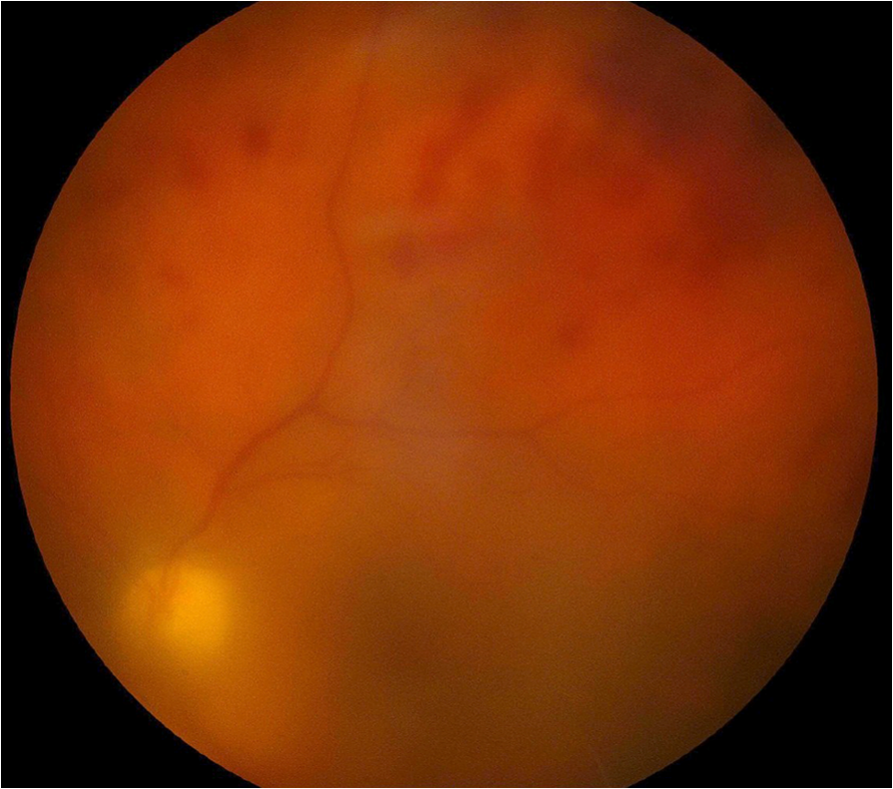
Left-eye fundus photo 1 week after the initial presentation with dense vitritis and retinal hemorrhages.

On presentation, his best-corrected visual acuity was 20/100 in the right eye and unobtainable in the left eye due to his altered mental status. The pupils were equal bilaterally with a brisk direct response and no relative afferent pupillary defect. His intraocular pressure was 5 mmHg bilaterally. Slit lamp exam revealed diffuse keratic precipitates and 4+ cell in both eyes. He had a 0.2-mm hypopyon in the right eye and a 0.4-mm hypopyon in the left eye. Dilated fundus exam showed 2+ to 3+ vitreous cell and 3+ haze, numerous intraretinal hemorrhages, and possible areas of diffuse retinitis along the vasculature (Figure 1).

Both eyes had anterior chamber and vitreous paracenteses followed by intravitreal injections of vancomycin, ceftazidime, and foscarnet were done. Initial testing on the ocular fluids was normal including gram stain and bacterial culture, fungal smear and culture, and polymerase chain reaction (PCR) for CMV, varicella zoster virus (VZV), herpes simplex virus (HSV), Epstein-Barr virus (EBV), and toxoplasma. The brain MRI was unremarkable. Cerebrospinal fluid (CSF) analysis showed an opening pressure of 21 cmH_2_O, four nucleated cells, glucose of 42 mg/dl, and total protein of 38 mg/dl. CSF bacterial and fungal cultures were negative as was the PCR testing for CMV, VZV, HSV, EBV, JC virus, Lyme disease, and toxoplasma. Real-time PCR assay for HHV-6 on the CSF was positive at 34.66 DNA target copies/μl. PCR reanalysis of the aqueous and vitreous samples detected HHV-6 in both vitreous samples (37.39 DNA target copies/μl in the right eye and 36.12 DNA target copies/μl in the left eye). The aqueous samples were negative for HHV-6.

**Figure 2:**
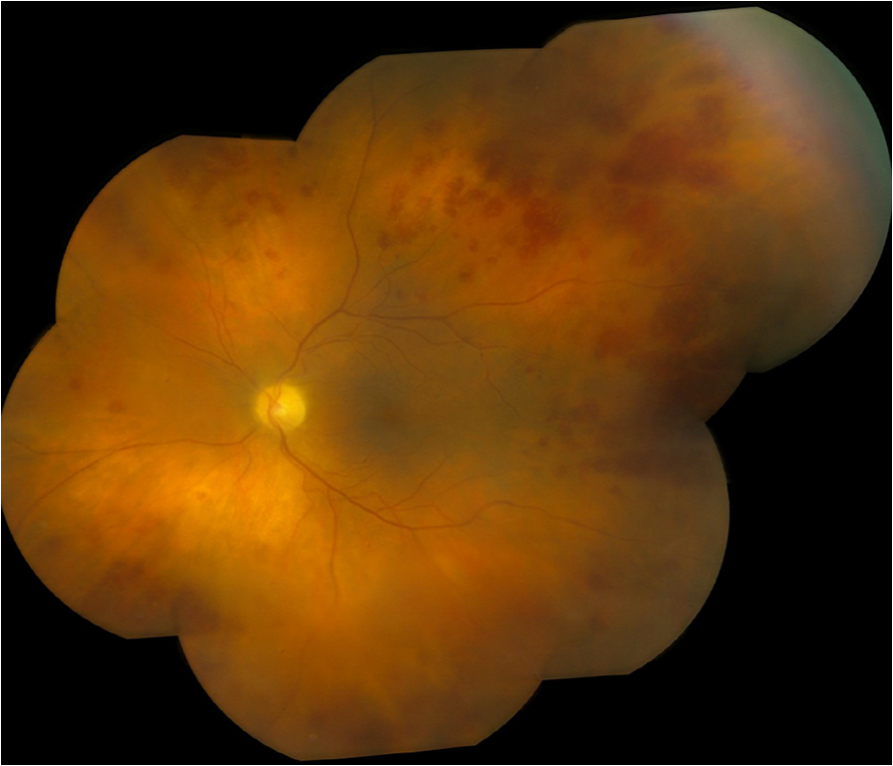
**Left-eye color montage photograph 2 weeks after the initial presentation with improved vitritis.** Intraretinal hemorrhages and diffuse retinal opacification can be appreciated.

The patient was treated with intravenous (IV) ganciclovir 5 mg/kg every 12 h for 1 month. Both eyes received twice-weekly intravitreal injections of foscarnet for 3 weeks, followed by weekly injections for 6 weeks. The total number of injections was 12 in each eye. Two weeks after presentation, his visual acuity had improved to 20/50 in both eyes, and the vitritis was significantly improved. He still had multiple intraretinal hemorrhages, but the areas of retinal whitening had not become necrotic (Figure 2). One month after the initial presentation, his visual acuity was 20/20 in the right eye and 20/25 in the left eye, and the retinal hemorrhages continued to improve.

After a month of intravenous treatment, HHV-6 DNA was still detectable in his serum by PCR, so the patient was switched to IV foscarnet due to the concern for ganciclovir resistance. Clinically, the encephalitis resolved, and the uveitis improved; however, after 7 weeks of intravenous antiviral treatment, the HHV-6 serum PCR remained positive for HHV-6 (>two million copies/ml). After several weeks on foscarnet, his renal function worsened, and he developed a lower extremity rash that was diagnosed as leukocytoclastic vasculitis on the basis of a biopsy. Due to the extremely high HHV-6 copy number, chromosomal integration of HHV-6 was suspected, and IV foscarnet was discontinued due to systemic complications. He received a total of 7 weeks of intravenous antiviral treatment.

**Figure 3:**
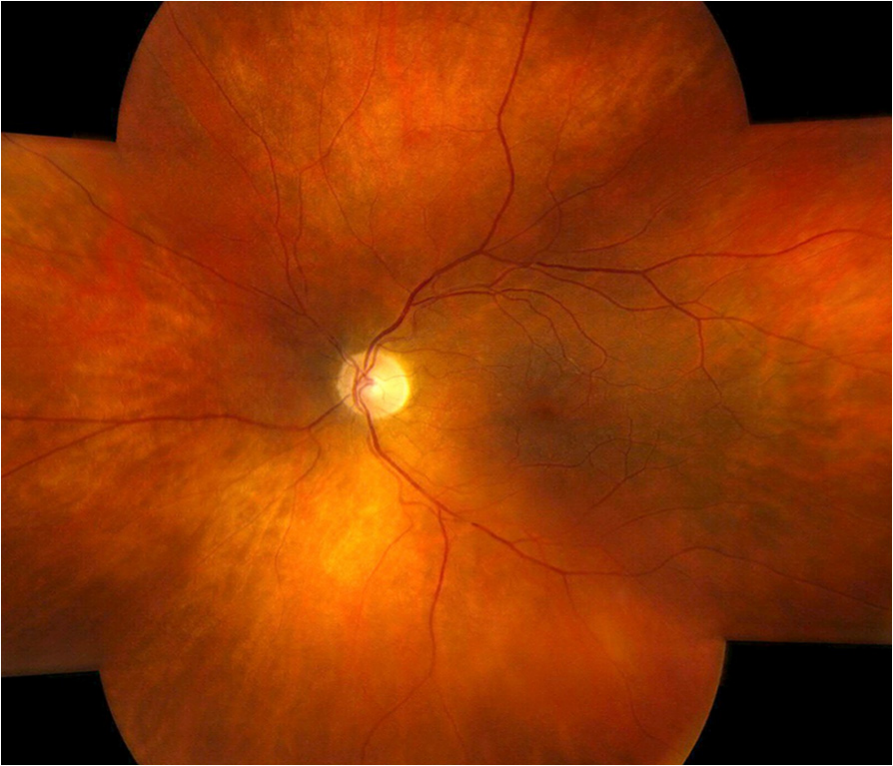
Left-eye color montage 6 months after the initial presentation showing resolution of vitritis, intraretinal hemorrhages, and retinitis.

A month later, he was readmitted due to fevers. After an extensive evaluation for infectious etiologies, IV ganciclovir was resumed with subsequent clinical improvement. He did not have any new visual symptoms. Six months after presentation, his visual acuity was 20/20 in the right eye and 20/30 in the left eye with complete resolution of the retinal hemorrhages and no evidence of active panuveitis (Figure 3).

### Discussion

HHV-6 infection is widespread with 95% seroprevalence in adults [1,9]. Of the two distinct variants, HHV-6A and HHV-6B, HHV-6B causes the majority of symptomatic primary infections in childhood including roseola infantum [10]. Like all members of the Herpesviridae family, the virus remains latent in various cell types, especially mononuclear cells. Reactivation later in life has been associated with encephalitis and other neurologic sequelae [10].

There are very few reports of uveitis associated with HHV-6. Maslin and colleagues described an 81-year-old immunocompromised man with bilateral intermediate uveitis and confirmed the presence of HHV-6 in aqueous humor and CSF by PCR analysis [5]. He was successfully treated with intravenous foscarnet and oral ganciclovir. Although HHV-6 was detected in his CSF, he did not have any neurologic symptoms, unlike our patient who had encephalitis. HHV-6A is more neurotropic than HHV-6B, so it is possible that the difference in the clinical presentation is related to infection with different variants; PCR analysis did not differentiate between the variants in our patient. Two additional cases of unilateral intermediate and panuveitis were attributed to HHV-6; however, other pathogens were also detected in the ocular fluids [6,7]. Most recently, a case of recurrent unilateral posterior uveitis due to HHV-6 was reported in an HIV-positive patient who was successfully treated with oral valacyclovir [8].

One unique aspect of our case was the development of HHV-6 chromosomal integration. Chromosomal integration occurs when the complete viral genome is incorporated into the host DNA. Although full-length viral DNA integration has been reported with another Herpesviridae family member, EBV, host chromosomal integration by HHV-6 is most consistently observed [1,11]. HHV-6 chromosomal integration is estimated to occur in approximately 1% of the world population, but the mechanism of integration has not yet been established [1,11]. Due to the germline integration, the virus can be vertically transmitted in a Mendelian manner and may also be transmitted via allogeneic hematopoietic stem cell transplantation [12]. Whole blood levels greater than 5.5 log_10_ DNA copies/ml are strongly suggestive of integration [12]. Among individuals who develop chromosomally integrated HHV-6, PCR testing can lead to misdiagnosis, unnecessary treatment, and difficulty determining disease resolution. Our patient underwent several weeks of intravenous and intravitreal antiviral treatments, despite clinical improvement, due to persistent HHV-6 positivity in his serum. In cases of viral DNA chromosomal integration, physicians must use clinical judgment to determine if the patient still has active HHV-6 infection instead of relying on serum viral levels as a measure of disease resolution.

We report this case to increase awareness of HHV-6 as a rare cause of panuveitis, especially in immunocompromised patients. Our patient differed from the previously reported cases since he developed encephalitis as well as bilateral panuveitis. In addition, our case is unique since HHV-6 chromosomal integration played an important role in the clinical management. If chromosomal integration occurs, PCR is an unreliable modality for monitoring treatment because the number of DNA copies does not necessarily correlate with active viral replication. Although HHV-6 is a rare cause of infectious uveitis, it is important to include this virus in the differential diagnosis of uveitis in an immunocompromised host. If the DNA copy number remains significantly elevated despite clinical improvement, HHV-6 chromosomal integration should be suspected.

## Competing interests

The authors declare that they have no competing interests.

## Authors’ contributions

JB designed and conducted the study and wrote the manuscript. WMS designed the study and assisted in writing the manuscript. Both authors read and approved the final manuscript.

## Disclosure

This paper is presented as a case report at the International Uveitis Study Group/Sociedad Panamericana de Enfermedades Inflamatorias Oculares Joint Meeting on November 2013 at New Orleans, LA, USA.
